# Effect of Bilateral Subthalamic Nucleus (STN) Deep Brain Stimulation (DBS) on drug reduction for Parkinson’s Disease: A retrospective observational study from Pakistan

**DOI:** 10.12669/pjms.40.12(PINS).11111

**Published:** 2024-12

**Authors:** Zubair Mustafsa Khan, Shahzeb Ahmad, Haseeb Mehmood Qadri, Ahtesham Khizar, Asif Bashir

**Affiliations:** 1 Dr. Zubair Mustafa Khan, MBBS, FCPS Assistant Professor Neurosurgery, Department of Neurosurgery Unit-I, Punjab Institute of Neurosciences, Lahore, Pakistan; 2 Dr. Shahzeb Ahmad, MBBS Postgraduate Resident Neurosurgery, Department of Neurosurgery Unit-I, Punjab Institute of Neurosciences, Lahore, Pakistan; 3 Dr. Haseeb Mehmood Qadri, MBBS Postgraduate Resident Neurosurgery, Department of Neurosurgery Unit-I, Punjab Institute of Neurosciences, Lahore, Pakistan; 4 Dr. Ahtesham Khizar, MBBS, FCPS Senior Registrar Neurosurgery, Department of Neurosurgery Unit-I, Punjab Institute of Neurosciences, Lahore, Pakistan; 5 Prof. Dr. Asif Bashir, MD, FAANS, FACS Professor of Neurosurgery, Department of Neurosurgery Unit-I, Punjab Institute of Neurosciences, Lahore, Pakistan

**Keywords:** Parkinson’s Disease, Deep Brain Stimulation, Subthalamic Nucleus, Unified Parkinson’s Disease Rating Scale, Dyskinesias, Levodopa

## Abstract

**Objectives:**

To determine the effect of Bilateral Subthalamic Nucleus (STN) Deep Brain Stimulation (DBS) on drug reduction for Parkinson’s disease (PD) in a low-middle-income country.

**Methods:**

This retrospective cohort study included 49 patients following interview based questionnaires who underwent bilateral STN DBS at the Department of Neurosurgery, Punjab Institute of Neurosciences, Lahore, Pakistan over five years (July 30, 2018 to June 29, 2023). Patients meeting the inclusion and exclusion criteria (49 patients) were selected and the effect of bilateral STN DBS on drug reduction was evaluated.

**Results:**

Following bilateral STN for Parkinsons Disease Levodopa equivalent daily dose (LEDD) and Unified Parkinson’s Disease Rating Scale (UPDRS)-III results were statistically significant, with a P-value of 0.0001. Effect of DBS on UPDRS-IV was 0.2751, which is statistically insignificant. LEDD reduced by 55.03% (P<0.0001), UPRS-III improved by 80.49% (P<0.0001), and UPDRS-IV improved by 1% (P<0.0001). Time spent with dyskinesia reduced by 17.54% (P<0.0001), whereas time spent off period reduced 22.44% (P<0.0001).

**Conclusion:**

When the disease is in its early stages and has not yet manifested advanced Parkinsons symptoms, bilateral STN DBS is an effective treatment option. It considerably reduces the need for levodopa and significantly improves the motor symptoms of rigidity, tremors, and bradykinesia.

**List of abbreviations:**
STN: Subthalamic nucleus,
DBS: Deep brain stimulation,
PD: Parkinson’s disease,
MSA: Multiple system atrophy,
PSP: Progressive supranuclear palsy,
CBD: Corticobasal degeneration,
UPDRS: Unified Parkinson’s Disease Rating Scale,
MMSE: Mini mental state examination,
MoCA: Montreal cognitive assessment,
LEDD: Levodopa equivalent daily dose,
PWP: People with Parkinson’s,
SEM: Standard error of mean,
CI: Confidence interval,
LMICs: Low and Middle Income Countries,
LEDD: Levo dopa equivalent daily dose,
UPDRS: Unified Parkinson’s Disease Rating Scale.

## INTRODUCTION

With advancing disease and duration of prevalence of clinical manifestations of Parkinson’s disease (PD), medications lose their efficacy and it becomes challenging to treat this neurodegenerative disease conservatively.^1^ The most commonly reported side effects of anti-Parkinsonian drugs are restless legs and dyskinesias, which make their utility short-termed and patients discontinue them.^2^ Deep brain stimulation (DBS) is arguably the most promising alternative treatment option available.^3^ It provides significant relief of motor symptoms and improves quality of life.^4^ There are some attributes of PD that remain virtually unaffected by stimulation. Precision of target area and number of leads affect the responsiveness of neuro-stimulation, and some symptoms are innately resistant to this treatment modality.^5^-^8^ DBS helps alleviate symptoms and provides major motor relief. It also decreases potency and dosing of essentially required drugs.^3^,^9^,^10^

DBS of bilateral subthalamic nucleus (STN) is proved to be effective in markedly improving quality of life. It is highly recommended in patients with 10 or more years of debilitating symptoms as well as in patients who are psychologically in-adjustable.^11^-^13^ Levodopa-responsive Parkinsonism responds to electrical stimulation.^14^

Believing the fact that DBS decreases drug dose and frequency; while improving patients’ quality of life, we conducted this study to compare preoperative drug requirements and their adverse effects, with post-DBS drug requirements and the benefits of bilateral STN DBS with respect to Unified Parkinson Disease Rating scale (UPDRS).

## METHODS

A retrospective observational study was conducted at the Department of Neurosurgery, Punjab Institute of Neurosciences (PINS), Lahore, Pakistan, over a period of five years (July 30, 2018 to June 29, 2023). This was a non-probability based consecutive case series of 49 patients.

### Ethical Approval:

Our study was exempted by institutional review board with reference number 1757/IRB/PINS/Approval/2024, dated March 6, 2024.

### Criteria for DBS Surgery in Parkinsons:

Following are the inclusion & exclusion criteria for DBS surgery in Parkinsons patient, at our institute:

### Inclusion criteria:

• PD with disease for three years or more
• Age: more than 15 years
• Responsive to levodopa
• On & off phenomenon
• Normal brain imaging
• On LEDD for at least three years

### Exclusion criteria:

• Parkinsonism with features of multiple system atrophy (MSA), progressive supranuclear palsy (PSP), and corticobasal degeneration (CBD).
• Dementia
• Articulation and swallowing problems
• Non-responsive to levodopa
• Impaired cognition and balance
• Significant psychological disorder

### Data collection procedure:

The study was launched after receiving approval from the institutional review board. All patients who underwent bilateral STN Deep brain stimulation for Parkinsons disease at Department of Neurosurgery Unit-I, Punjab Institute of Neurosciences, Lahore, Pakistan, were included in the study. Every participant provided informed consent to be enrolled in our study. Preoperative data were reviewed, and postoperative follow-up was obtained directly from patients. We enrolled 49 individuals in this study, two died, for a total of 51 patients. This was the experience of a single team. We tracked the preoperative and postoperative features of the patients, who were all administered neurostimulation based on inclusion criteria. To measure psychosocial incapacity, the Mini Mental State Examination (MMSE) and the Montreal Cognitive Assessment (MoCA) tests were obtained from patient record. Pre-operative medication dosages, motor examination (UPDRS-III) and dyskinesia scores (UPDRS-IV), as well as the time spent with dyskinesia and the off period (no response to drugs) were obtained from patient record, whereas post operative data were directly obtained from patient. Complications were also mentioned.

### DBS Procedure:

All patients planned for surgery undergo DBS protocol MRI a day before surgery. The surgical planning is done a day before that includes target, entry and trajectory. On day of surgery stereotactic frame (Leksell G) is placed under local under anaesthesia and CT Brain plain is done. The CT scan data is loaded and merged with already planned MRI of patient using Framelink software. First stage of surgery is performed under local anaesthesia during which each cranial lead of DBS is implanted along already planned trajectory following MER and macrostimulation of target. The effetcs and side effects are noted before final placement of leads. After completion of first stage second stage surgery is performed under general anaesthesia that includes attachment of extensions, tunnelling and implantation of IPG. Connections are verified by testing impedence of system. Deep Brain stimulation is turned on after one week of surgery.

### Data analysis procedure:

The data was analyzed using GraphPad Prism 8.02. Frequency and percentages were reported for categorical variables as shown in Table-I. Central tendencies and dispersion were shown for numerical variables. Paired t-tests were performed to assess pre- and post-operative medication doses, UPDRS-III, UPDRS-IV, time spent with dyskinesia, and off period, and outliers were treated accordingly. All the medications were converted to LEDD (levodopa equivalent daily dose) with the help of a calculator by people with Parkinson’s (PWP).

**Table-I:** Parameters of patients in the study

S. No.	Parameters		Frequency
1	Mean age (years)		56.79 ± 1.825 SD
2	Gender	Male	41 (80%)
3		Female	8 (20%)
4	Mean disease duration (years)		10.12 ± 0.467 SD
5	Mean preoperative LEDD		1187 ± 404.1 SD
6	Mean postoperative LEDD		533.7 ± 319.7 SD
7	Mean preoperative UPDRS-III		28.55 ± 11.35 SD
8	Mean postoperative UPDRS-III		5.571 ± 6.627
9	Mean preoperative UPDRS-IV		9.122 ± 3.695
10	Mean postoperative UPDRS-IV		8.979 ± 2.654

### Endpoint measurement:

Primary endpoint was defined as drug reduction post-DBS; this was calculated by LEDD and PWP. LEDD was calculated pre operatively and compared to the requirement for medicine when neurostimulation was performed on each patient individually. Following a substantial result for drug reduction, we estimated clinically relevant motor fluctuations (UPDRS-III) and clinical adverse effects of medication (UPDRS-IV) as main secondary outcomes. Major outcomes were time spent with dyskinesia and time off. To rule out cognitive impairment and severe dementia, we employed a MoCA test score of less than 22. We also employed the MMSE as a minor secondary outcome to remove individuals from the study who were severely impaired. We experienced a negligible requirement for electrical stimulation within seven days of surgery (lesioning effect). Maximum results were obtained in one month on average. There is no minimum and maximum dose of drugs. We give according to side effect profile that is why we calculate Levo dopa equivalent daily dose (LEDD) for the patient is on multiple drugs.

### Adverse events:

We defined major adverse events as any incident that required the patient to be readmitted to the hospital or resulted in the patient’s death. One patient’s implant got infected and required reoperation for implant revision.

### Statistical analysis:

**Fig.1:**
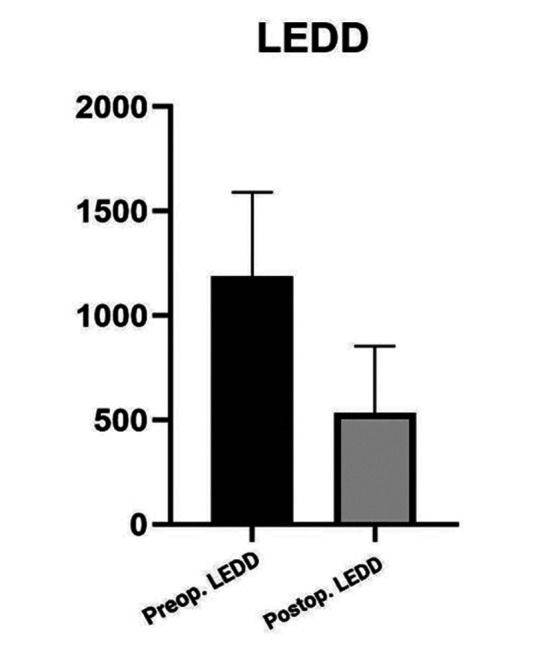
Preoperative and postoperative LEDD.

**Fig.2:**
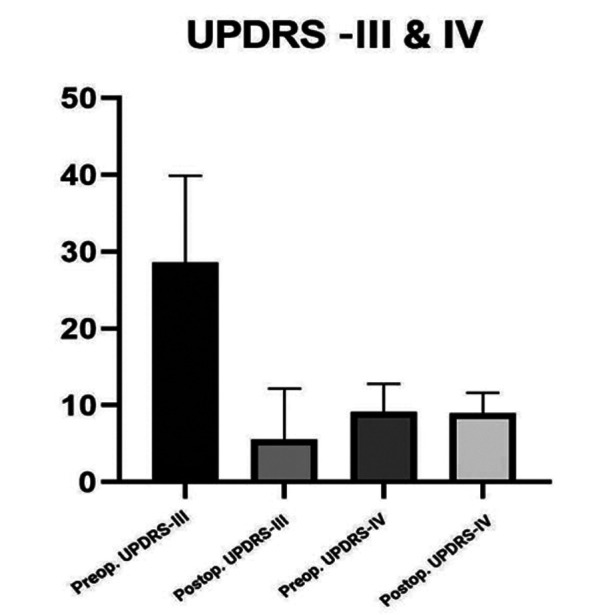
Preoperative and postoperative UPDRS-III & IV.

Sampling was sequentially convenient. We chose a 95% CI (confidence interval). The means were compared using paired t-tests. Wilcoxon signed rank tests were employed to treat the data’s outliers, which included two extremely low and one extremely high value. The difference in pre- and post-operative LEDD means was computed as shown in Fig.1. In addition, the pre- and post-operative UPDRS-III and IV means were compared as given in Fig.2.

## RESULTS

**Fig.3:**
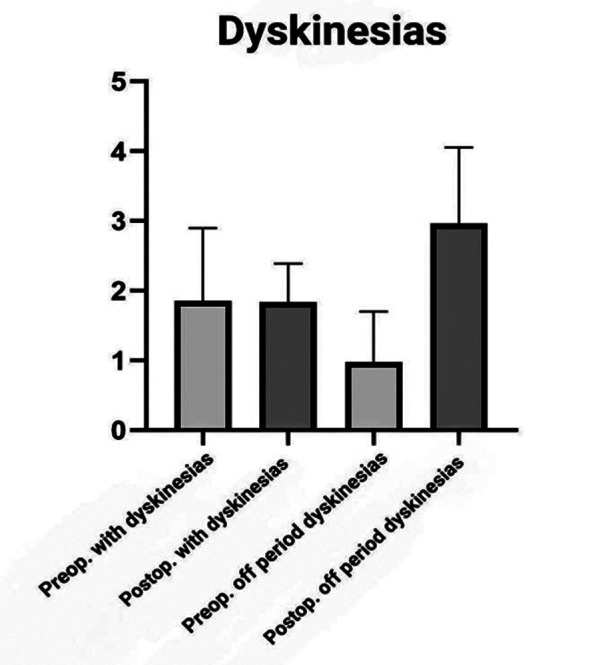
Preoperative and postoperative period with dyskinesias and off dyskinesias.

LEDD and UPDRS-III results were statistically significant, with a P-value of 0.0001 as shown in Table-II. For UPDRS-IV, it was 0.2751, which is statistically insignificant. LEDD improved by 55.03% (P<0.0001), UPRS-III improved by 80.49% (P<0.0001), and UPDRS-IV improved by 1% (P<0.0001). Time spent with dyskinesia means percentage of time per 24 hours in which patient experienced dyskinesias decreased by 17.54% (P<0.0001), whereas time spent off means percentage of the time per day spent without dyskinesias increased by 22.44% (P<0.0001) as shown in Fig.3.

**Table-II:** Pre and post operative UPDRS.

S. No.	Age (years)	Gender	Duration of disease before DBS (years)	Pre operative LEDD	Post operative LEDD	Pre operative UPDRS III	Post operative UPDRS III	Pre operative UPDRS IV	Post operative UPDRS IV
1	49	Male	14	1210	440.0	31.00	14.00	12.00	5.00
2	49	Male	7	1215	395.0	55.00	2.00	15.00	13.00
3	73	Female	15	1460	740.0	46.00	10.00	11.00	8.00
4	46	Male	8	1310	125.0	38.00	4.00	11.00	11.00
5	35	Male	8	1200	340.0	37.00	6.00	8.00	7.00
6	39	Male	7	1060	600.0	23.00	2.00	10.00	10.00
7	87	Male	14	1210	700.0	40.00	.00	8.00	8.00
8	35	Male	14	1010	125.0	6.00	.00	3.00	8.00
9	55	Female	10	1200	405.0	55.00	8.00	16.00	8.00
10	38	Female	11	1210	553.0	36.00	2.00	7.00	9.00
11	25	Male	7	1000	240.0	41.00	7.00	8.00	14.00
12	65	Male	11	5	475.0	22.00	3.00	8.00	7.00
13	68	Female	7	1560	385.0	50.00	21.00	5.00	12.00
14	58	Male	8	1110	455.0	39.00	7.00	19.00	9.00
15	87	Male	14	1210	700.0	40.00	.00	8.00	8.00
16	55	Female	11	1700	62.5	24.00	6.00	14.00	6.00
17	64	Male	15	1265	290.0	40.00	1.00	9.00	3.00
18	69	Male	5	1710	1560.0	30.00	10.00	11.00	8.00
19	69	Female	10	1710	240.0	19.00	3.00	7.00	12.00
20	59	Male	13	2157	595.0	24.00	.00	16.00	10.00
21	58	Male	13	1205	960.0	36.00	4.00	12.00	7.00
22	46	Male	12	1010	265.0	18.00	2.00	10.00	8.00
23	46	Male	15	1170	400.0	26.00	4.00	5.00	8.00
24	63	Male	9	1290	840.0	40.00	30.00	14.00	14.00
25	64	Male	10	1210	575.0	21.00	12.00	10.00	15.00
26	47	Male	12	465	440.0	20.00	4.00	10.00	9.00
27	70	Male	5	910	640.0	36.00	.00	14.00	11.00
28	68	Male	3	0	790.0	17.00	4.00	1.00	9.00
29	41	Male	10	1225	895.0	6.00	1.00	6.00	8.00
30	69	Male	17	1090	500.0	18.00	27.00	9.00	10.00
31	58	Male	12	1700	0.0	32.00	5.00	13.00	4.00
32	49	Male	9	1060	1590.0	32.00	8.00	8.00	7.00
33	57	Male	10	1240	495.0	26.00	4.00	7.00	10.00
34	56	Female	9	1240	485.0	24.00	3.00	7.00	8.00
35	57	Male	8	1365	475.0	22.00	1.00	8.00	9.00
36	60	Male	9	1240	475.0	20.00	.00	8.00	6.00
37	50	Male	10	1210	350.0	23.00	1.00	6.00	7.00
38	59	Male	9	1490	390.0	24.00	3.00	6.00	9.00
39	57	Male	10	1460	415.0	17.00	4.00	7.00	8.00
40	58	Male	12	1240	740.0	23.00	8.00	6.00	8.00
41	70	Male	5	910	595.0	36.00	.00	14.00	10.00
42	68	Male	3	0	960.0	17.00	4.00	3.00	7.00
43	41	Male	10	1225	265.0	6.00	2.00	6.00	8.00
44	69	Male	17	1090	400.0	18.00	4.00	8.00	8.00
45	58	Male	12	1700	840.0	32.00	30.00	13.00	14.00
46	49	Male	9	1060	575.0	32.00	12.00	7.00	15.00
47	57	Male	10	1240	440.0	26.00	4.00	7.00	8.00
48	56	Female	9	1240	640.0	24.00	.00	7.00	11.00
49	57	Male	8	1365	790.0	22.00	4.00	8.00	9.00

**Table-III:** Mean major outcomes with percentage improvements.

Primary endpoint	Medications only (Mean ± SD)	Neurostimulation + medications (Mean ± SD)	Difference between means ± SEM	P-value	Percentage improvements (%)
LEDD	1187 ± 404.1	533.7 ± 319.7	653.3 ± 73.61	<0.0001	55.03
UPDRS-III	28.55 ± 11.35	5.571 ± 6.627	22.98 ± 1.87	<0.0001	80.49
UPDRS-IV	9.122 ± 3.695	8.979 ± 2.654	0.143 ± 0.65	0.2751	1
Time spent with dyskinesia	1.857 ± 1.041	0.9796 ± 0.7214	0.877 ± 0.18	<0.0001	17.54
Time spent with off period	1.837 ± 0.5533	2.959 ± 1.098	1.122 ± 0.1757	<0.0001	22.44

## DISCUSSION

Our center is the only public-sector hospital offering DBS for the patients of PD in Pakistan. People are not aware of neuro-stimulation and its effects on drug reduction. Efforts are being made to create the awareness of DBS in Pakistan gradually. The mean age at presentation in our center is 56.79 ± 1.825 years, which is comparable to a mean age of 59.1 ± 2.9 years as highlighted by the recent meta-analysis of Lachenmayer and colleagues.^12^

Bove et al. conducted an observational study showing long term outcomes in bilateral STN DBS in 51 patients with PD, with a mean of 17.06 ± 2.18 years onset of disease. They found 50.6% drug reduction as compared to our study showing 55.03% drug reduction. Time spent with dyskinesia improved by 75% while the time spent with dyskinesia improved by 17.54% in our experience. Time spent with off period reduced by 58.7% in their study while our showed 22.44% improvement.^15^ Obeso et al showed 96 patients with LEDD reduction 38% (p < 0.001) marginally behind our study showing 55.03% (p < 0.001), 62% reduction in off time and 70% dyskinesia reduction at 6 months visit after DBS.^16^

Deuschl et al. conducted a multicentric, unblinded trial comparing STN DBS with best medical management for patients under 75 years of age with idiopathic PD. Their study demonstrated UPDRS-III improvement of 39.3% improvement with medications stopped while 67.7% off-time reduction. While our research highlights an 80.49% UPDRS-III improvement and 22.4% off-time reduction.^17^

Schuepbach et al. conducted a study of 24 months duration comparing the group of neuro-stimulation plus medical therapy and medical therapy alone, including the patients with mean age of 52 years. They concluded 49.1% UPDRS-III improvement for patients managed by neuro-stimulation. About 73.4% of patients who were on neuro-stimulation plus medical therapy had UPDRS-IV improvement and 65.9% had LEDD reduction. In our study, 1% patients had UPDRS-IV improvement and 55.03% LEDD reduction with drugs and neurostimulation.^18^

Kalhoro and Hashim conducted a study including 21 patients showing similar results except hypophonia and postural instability resistant to improvement.^19^ Mahmood et al. also treated 44 patients and showed an improvement in UPDRS (part-III) score from 45.19 ± 0.90 at baseline to 25.15 ± 1.20 at the end of 3^rd^.^20^

### Limitations:

Our study’s weaknesses were mostly due to its retrospective approach, limited sample size, and shorter follow-up duration. Another difficulty with a low-middle-income country is a lack of movement disorder specialists and other programming team members.

### Recommendations:

Longitudinal studies should be conducted in subsequent studies to investigate the repercussions over time.

## CONCLUSIONS

We found that bilateral STN DBS is an effective option for treating Parkinsons disease with debilitating symptoms. It dramatically improves motor symptoms such as rigidity, tremors, and bradykinesia while also lowering the need for levodopa.

### Authors’ Contribution:

**ZMK:** Conceived the idea, designed the study and Critical review of manuscript

**SA:** Data acquisition, Data analysis and Manuscript drafting.

**HMQ and AK:** Literature search and review, Manuscript drafting and Interpretation of results.

**AB:** Critical review of manuscript and supervision.

All authors have read and approved the final manuscript and are responsible for integrity of research.

## References

[1] Szász JA, Constantin VA, Orbán-Kis K, Rácz A, Bancu LA, Georgescu D (2019). Profile of Patients With Advanced Parkinson's disease Suitable For Device-Aided Therapies:Restrospective Data Of A Large Cohort Of Romanian Patients. Neuropsychiatr Dis Treat.

[2] Nakanishi E, Takahashi R (2019). Side Effects, Contraindications, and Drug-Drug Interactions in the Use of Antiparkinsonian Drugs. NeuroPsychopharmacotherap.

[3] Bove F, Mulas D, Cavallieri F, Castrioto A, Chabardès S, Meoni S (2021). Long-term Outcomes (15 Years) After Subthalamic Nucleus Deep Brain Stimulation in Patients with Parkinson Disease. Neurology.

[4] Dannug AT, Gabriel FGC, Macias MCYL, Diesta CCE (2021). Impact of deep brain stimulation on quality of life and motor symptoms in Parkinson's disease and X-linked dystonia parkinsonism:The Philippine experience. Parkinsonism Relat Disord.

[5] Wilkins KB, Parker JE, Bronte-Stewart HM (2020). Gait variability is linked to the atrophy of the Nucleus Basalis of Meynert and is resistant to STN DBS in Parkinson's disease. Neurobiol Dis.

[6] Reddy DA, Rama Raju V, Narsimha G (2021). Deep Brain Stimulation Coding in Parkinson's:An Evolving Approach. IETE J Res.

[7] Pintér D, Járdaházi E, Balás I, Harmat M, Makó T, Juhász A (2023). Antiparkinsonian Drug Reduction After Directional Versus Omnidirectional Bilateral Subthalamic Deep Brain Stimulation. Neuromodulation.

[8] Hoffman-Ruddy B, Schulz G, Vitek J, Evatt M (2001). A preliminary study of the effects of sub thalamic nucleus (STN) deep brain stimulation (DBS) on voice and speech characteristics in Parkinson's Disease (PD). Clin Linguist Phon.

[9] Liu FT, Lang LQ, Yang YJ, Zhao J, Feng R, Hu J (2019). Predictors to quality of life improvements after subthalamic stimulation in Parkinson's disease. Acta Neurol Scand.

[10] Jost WH, Brück C (2002). Drug interactions in the treatment of Parkinson's disease. J Neurol.

[11] Weiss D, Volkmann J, Fasano A, Kühn A, Krack P, Deuschl G (2021). Changing Gears - DBS For Dopaminergic Desensitization in Parkinson's Disease?. Ann Neurol.

[12] Lachenmayer ML, Mürset M, Antih N, Debove I, Muellner J, Bompart M (2021). Subthalamic and pallidal deep brain stimulation for Parkinson's disease-meta-analysis of outcomes. NPJ Parkinsons Dis.

[13] Liu Y, Li F, Luo H, He Q, Chen L, Cheng Y (2019). Improvement of Deep Brain Stimulation in Dyskinesia in Parkinson's Disease:A Meta-Analysis. Front Neurol.

[14] Zheng Z, Yin Z, Zhang B, Fan H, Liu D, Zhou Y (2021). Levodopa Challenge Test Predicts STN-DBS Outcomes in Various Parkinson's Disease Motor Subtypes:A More Accurate Judgment. Neural Plast 2021.

[15] Bove F, Mulas D, Cavallieri F, Castrioto A, Chabardès S, Meoni S (2021). Long-term Outcomes (15 Years) After Subthalamic Nucleus Deep Brain Stimulation in Patients With Parkinson Disease. Neurology.

[16] Obeso JA, Olanow CW, Rodriguez-Oroz MC, Krack P, Kumar R, Lang AE, Deep-Brain Stimulation for Parkinson's Disease Study Group (2001). Deep-brain stimulation of the subthalamic nucleus or the pars interna of the globus pallidus in Parkinson's disease. N Engl J Med.

[17] Deuschl G, Schade-Brittinger C, Krack P, Volkmann J, Schäfer H, Bötzel K (2006). A randomized trial of deep-brain stimulation for Parkinson's disease. N Engl J Med.

[18] Schuepbach WM, Rau J, Knudsen K, Volkmann J, Krack P, Timmermann L (2013). Neurostimulation for Parkinson's disease with early motor complications. N Engl J Med.

[19] Kalhoro A, Hashim ASM (2023). Effectiveness of deep brain stimulation in Parkinson's disease treatment with Single-center experience in Pakistan. Pak J Med Sci.

[20] Mahmood K, Ali OA, Ali I (2020). Outcome of Deep Brain Stimulation (DBS) for the Treatment of Parkinson's Disease in Terms of Improvement in MDS-UPDRS Scale Over 5 Years. Pak J Neurolog Surg.

